# Unlocking the Genetic Secrets of Acromegaly: Exploring the Role of Genetics in a Rare Disorder

**DOI:** 10.3390/cimb46080538

**Published:** 2024-08-20

**Authors:** Ioana Balinisteanu, Lavinia Caba, Andreea Florea, Roxana Popescu, Laura Florea, Maria-Christina Ungureanu, Letitia Leustean, Eusebiu Vlad Gorduza, Cristina Preda

**Affiliations:** 1 Endocrinology Department, “Grigore T. Popa” University of Medicine and Pharmacy, 700115 Iasi, Romania; dr.balinisteanu.ioana@gmail.com (I.B.); mariachristina1105@gmail.com (M.-C.U.); letitialeustean@yahoo.com (L.L.); cristina.preda@umfiasi.ro (C.P.); 2 Medical Genetics Department, “Grigore T. Popa” University of Medicine and Pharmacy, 700115 Iasi, Romania; andreeaflorea97@gmail.com (A.F.); roxana.popescu@umfiasi.ro (R.P.); eusebiu.gorduza@umfiasi.ro (E.V.G.); 3 Nephrology-Internal Medicine Department, “Grigore T. Popa” University of Medicine and Pharmacy, 700115 Iasi, Romania; lflorea68@yahoo.com

**Keywords:** acromegaly, genetics, PitNET, AIP, FIPA, GNAS, MEN1, GPR101, Carney, McCune–Albright

## Abstract

Acromegaly is a rare endocrine disorder characterized by the excessive production of growth hormone (GH) in adulthood. Currently, it is understood that certain pituitary neuroendocrine tumors (PitNETs) exhibit a hereditary predisposition. These tumors’ genetic patterns fall into two categories: isolated and syndromic tumors. The isolated forms are characterized by molecular defects that predispose exclusively to PitNETs, including familial isolated pituitary adenomas (FIPAs) and sporadic genetic defects not characterized by hereditary predisposition. All the categories involve either germline or somatic mutations, or both, each associated with varying levels of penetrance and different phenotypes. This highlights the importance of genetic testing and the need for a more comprehensive view of the whole disease. Despite the availability of multiple treatment options, diagnosis often occurs after several years, and management is still difficult. Early detection and intervention are crucial for preventing complications and enhancing the quality of life for affected individuals. This review aims to elucidate the molecular, clinical, and histological characteristics of GH-secreting PitNETs, providing insights into their prevalence, treatment nuances, and the benefits of genetic testing for each type of genetic disorder associated with acromegaly.

## 1. Introduction

Acromegaly is a rare endocrine disorder characterized by the excessive production of growth hormone (GH) in adulthood, typically (approximately 95% of the time) due to a benign tumor in the pituitary gland, the so-called GH-secreting pituitary neuroendocrine tumor (PitNET). These tumors account for approximately 9–13% of all pituitary adenomas. Rarely, acromegaly can be caused by pituitary hyperplasia, especially associated with syndromic diseases like Carney complex, McCune–Albright syndrome (MAS), or X-linked acrogigantism. Extremely rarely, acromegaly can be due to extra-pituitary pathologies like hypothalamic adenomas or paraneoplastic ectopic GH secretion [1,2,3,4].

According to Crisafulli, Luxi et al., who published the first systematic review and meta-analysis of epidemiological findings in acromegaly in 2021, the global prevalence of acromegaly is 5.9 per 100,000 people, while the incidence is 0.38 cases per 100,000 person-years [5].

This overproduction of GH leads to an abnormal increase in the size of body tissues, particularly the bones and soft tissues, resulting in enlarged extremities and facial features. Acromegaly can cause various health complications, including hypertension, cardiovascular disease, diabetes mellitus, sleep apnea, and colonic polyposis [6].

Diagnosis is typically suspected based on distinctive clinical features or the identification of associated complications. It is then confirmed through elevated levels of IGF-1 (insulin-like growth factor 1) and by measuring unsuppressed GH during a glucose stimulation test. The average diagnostic delay is 5.5 years, reaching up to ≥10 years in 24% of the patients. Longer diagnosis delays correlate with a greater number of comorbidities [7].

In 2018, Bolfi, Neves et al. published a metanalysis regarding mortality in acromegaly, which concluded that mortality decreases in parallel with the achievement of biochemical control. When somatostatin analogs (SAs) were administered as adjuvant treatment, mortality in acromegaly was not elevated, but when patients were treated with surgery and/or radiotherapy, mortality was significantly higher. The use of SAs as adjuvant therapy has reduced mortality in recent years. However, the improved life expectancy has been accompanied by a rise in cancer-related deaths, a pathology that emerged as a leading cause of death among acromegaly patients [8].

Pathologically, somatotropinomas are divided into two groups based on the cytokeratin immunostaining pattern: sparsely granulated adenomas, which are known to be more aggressive and frequently resistant to first-line treatment options, and densely granulated adenomas, which present a milder evolution and favorable outcomes after therapy. Regarding immunohistochemistry, SSR2 (somatostatin receptor type 2)-negative tumors are typically resistant to first-generation analogs, whereas the presence of SSR5 (somatostatin receptor type 5) may be a prognostic sign for a good response to Pasireotide [9,10].

Early diagnosis and treatment are crucial in preventing further complications and improving quality of life for affected individuals. Treatment options include surgery, medication, and radiation therapy, often used in combination, to manage symptoms and normalize hormone levels. Regarding medical treatment, three classes of drugs are available: dopamine agonists such as Cabergoline, the GH receptor antagonist Pegvisomant, and somatostatin receptor agonists (first-generation Sas-Octreotide and Lanreotide, which target SSR2 in particular, and the new-generation Pasireotide, which has higher affinity to SSR5 than to SSR2) [11].

## 2. Isolated Acromegaly

**Figure 1:**
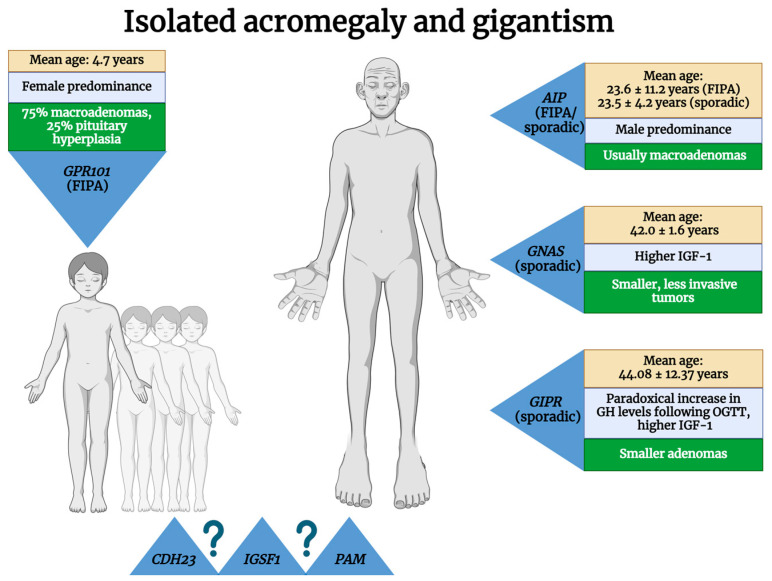
Isolated acromegaly and gigantism. Abbreviations: *GPR101*—G protein-coupled receptor 101; FIPA—familial isolated pituitary adenoma; *AIP*—aryl hydrocarbon receptor-interacting protein; *GNAS*—GNAS complex locus; IGF-1—insulin-like growth factor I; *GIPR*—gastric inhibitory polypeptide receptor; GH—growth hormone; OGTT—oral glucose tolerance test; *CDH23*—cadherin-related 23; *IGSF1*—immunoglobulin superfamily member 1; *PAM*—peptidylglycine alpha-amidating monooxygenase. The mean age refers to the average age at which acromegaly/gigantism occurs. The sex prevalence and pituitary anomalies refer to PitNETs. Created with BioRender.com (accessed on 14 August 2024).

In the context of genetics, acromegaly is classified into isolated and syndromic forms. A brief description of these classifications can be found in Table 1. Isolated acromegaly refers to molecular defects that exclusively predispose individuals to PitNETs, including familial isolated pituitary adenomas (FIPAs) and sporadic defects without a hereditary predisposition. An overview of the genes involved in isolated acromegaly is presented in Figure 1. In contrast, syndromic forms include acromegaly among other clinical particularities or diseases. As the name suggests, germline mutations are transmitted from parents to children and can be identified in germ cells. In contrast, sporadic mutations are acquired throughout an individual’s lifetime and are not inherited by their descendants [12]. Any change that occurs at the cellular level in somatic tissues following fertilization is referred to as a somatic mutation. Since these mutations do not affect the germline, they are not inherited by descendants. Throughout an organism’s life cycle, somatic mutations happen naturally because of errors in DNA repair mechanisms or as a direct reaction to stress. These mutations are a normal aspect of aging, but the mutations that occur early in embryonic development can alter the organism’s development by causing mosaicism within the gene line [13].

### 2.1. Familial Isolated Pituitary Adenomas—FIPAs

The term “familial isolated pituitary adenomas” (FIPAs) describes a medical condition where PitNETs, either secreting or nonsecreting, develop in two or more individuals within a family without any other clinical features, therefore they cannot be included in a syndrome. FIPAs account for 2–4% of all PitNETs [90]. Among FIPA families, 40% are homogenous (they have the same type of tumor), and 60% are heterogeneous (an association of cases of prolactinomas, acromegaly, and nonfunctioning adenomas). Acromegaly is responsible for 58% of homogeneous FIPAs, being the main type of PitNET in such families. The most common heterogeneous FIPA phenotype blends acromegaly and prolactinomas among the affected individuals (42%). In total, there is an elevated prevalence of somatotropinomas in FIPAs (35%), contrasting with general epidemiological findings ranging from 13% to 15% [91].

#### 2.1.1. Aryl Hydrocarbon Receptor-Interacting Protein (AIP)

1. General information: *AIP* is considered a tumor-suppressor gene. *AIP* mutations are found in PitNETs, like somatotroph, somatolactotroph, or lactotroph adenomas [31,92]. The transmission is autosomal dominant, with a limited penetrance of 20–33% [15,23,93].

2. Prevalence: Approximately 10–15% of FIPA families harbor germline heterozygous inactivating mutations in the *AIP* gene (loss of function) [14,15].

3. Clinical features: *AIP* mutations are correlated with a slight male predominance and a younger age of onset (≤18 years in 65% and <30 years in 87%) [16,18]. The median age at diagnosis is 23 years [17], and the mean age is 23.6 ± 11.2 years [23]. The symptoms tend to appear 8 years earlier, and the age at diagnosis is also 6 years earlier compared to patients with acromegaly who do not carry *AIP* mutations. The IGF-1 levels at diagnosis show no particularities. Tumors are usually macroadenomas at diagnosis, with suprasellar extension and a higher rate of apoplexy [16,18].

4. Histological findings: *AIP* mutations are associated with sparsely granulated GH-secreting adenomas. There are no densely granulated tumors described [19]. SSR2 concentration is reduced in *AIP*-mutated somatotropinomas [20].

5. Genetics: The *AIP* gene is located on chromosome 11q13.2; it consists of six exons, and it encodes a 330-amino-acid co-chaperone protein. Aryl hydrocarbon receptor (AHR)-interacting protein is widely expressed in tissues, and it is part of a multiprotein complex (AIP–AHR–heat shock protein 90 (HSP90) complex) [10,22,94]. The protein consists of a C-terminal domain with three tetratricopeptide repeats (TRPs), which regulates protein–protein interactions, and an N-terminal domain that is responsible for conserving the structure and stability of AIP and also plays a role in interactions with other proteins [95]. The AHR signaling pathway contributes to ligand-independent functions, such as the regulation of cell growth and differentiation [96]. In the context of pituitary adenomas, *AIP* acts as a tumor-suppressor gene. Mutant proteins lose their ability, or have a reduced ability, to block cell proliferation [19]. AIP also interacts with phosphodiesterase 4 (PDE4)-dependent protein kinase A (PKA) pathway activity. In mutant *AIP*s, the binding between AIP and its interacting partner, PDE4A5, is disturbed, leading to the increased modulation of cyclic adenosine monophosphate (cAMP) [19,96]. The cAMP pathway is crucial in endocrine tumors, especially in the secretory ones, as it influences hormone production and release, thereby regulating cellular functions and growth [95]. To date, more than 40 pathogenic variants mutations have been identified for the *AIP* gene, most of them being nonsense mutations, followed by frameshift ones. A substantial number of variants of uncertain significance (496) have also been reported [97]. The most frequent pathogenic variants are c.40C>T, p.Gln14Ter (Finnish founder variant), c.241C>T, p.Arg81Ter (mutational hotspot), c.805_825dup, p.Phe269_Hid275dup (European founder variant), c.910C>T, and p.Arg304Ter (the most common mutational hotspot in Irish, Romanian, English, Italian, Indian, and Mexican populations and a founder effect in Ireland) [17].

AIP-FIPA has an autosomal dominant inheritance with reduced penetrance. Each child of an individual with an *AIP* pathogenic variant has a 50% risk of inheriting the mutation. Prenatal testing is possible if the pathogenic variant is known, but it does not provide reliable forecasts regarding tumor development, adenoma type, age of onset, and prognosis. Because of the reduced penetrance, the parent of an affected individual may not be clinically affected, but in almost all cases, one parent is heterozygous for the *AIP* pathogenic variant. Possible explanations for the absence of the mutation in both parents are germline mosaicism in a parent or a de novo pathogenic variant in the child. In the case of germline mosaicism in a parent, the risk of inheritance in a sibling is 1%. Because of this, negative family history in a proband cannot be confirmed unless extensive molecular genetic testing is performed on the parents [17].

Diagnosis is established via the identification of a heterozygous germline pathogenic variant in *AIP* by molecular genetic testing, whether gene-targeted testing or comprehensive genomic testing. Firstly, sequence analysis (single-gene testing) should be performed and can detect small intragenic deletions/insertions and missense, nonsense, and slice-site variants [17].

6. Personalized treatment: There is a resistance to the first class of somatostatin analogs cited in the literature due to the low expression of SSR2. However, at longer follow-up, there is no difference in the rate of active disease, but this is probably due to the radiotherapy that *AIP*-positive tumors more often require. Repeat surgery and multimodal therapy are also noted [11,16,18].

7. Benefits of genetic testing: The PitNETs among the silent *AIP*-positive carriers (diagnosed by screening the relatives of *AIP*-positive patients with clinical manifestations) showed a better prognosis: smaller dimensions (microadenomas) and less associated with suprasellar extension and cavernous sinus invasion. The treatment is also manageable with fewer interventions (medical or operative), and no radiotherapy is needed. The rates of active disease and hypopituitarism are also reduced [16]. A proportion of 9.5–11.7% carriers were found with a PitNET, of which 36.4% were diagnosed as acromegaly, and 9.1% as gigantism [16,98]. The optimal duration for monitoring *AIP*-positive asymptomatic patients remains uncertain, as most individuals receive a diagnosis before the age of 30. For instance, a large study of 443 patients with apparently random pituitary adenomas found that only 3 out of 16 *AIP*-positive individuals began showing symptoms between the ages of 30 and 40. Moreover, no *AIP* mutations were detected in patients diagnosed after the age of 40. As a result, current recommendations suggest decreasing the frequency of screening after the age of 30 and discontinuing screening after 50 [31,99,100]. Another benefit of genetic testing is reducing the cost of screening family members that do not carry a pathogenic mutation [17].

#### 2.1.2. G Protein-Coupled Receptor 101 (GPR101)–X-Linked Acrogigantism (X-LAG)

1. General information: Microduplications on chromosome Xq26.3 are responsible for X-LAG, a subtype of gigantism that presents a very early onset. There are also recurrent mutations in *GPR101* in some adults with acromegaly [24]. It has been established that most mutations are germline. However, it is also important to note the presence of somatic mutations, which should be actively sought when clinical features are evident but no germline mutations are detected [25]. Even though the penetrance of the disease is 100%, most patients with X-LAG syndrome are sporadic (72.2%), probably due to de novo mutations [26].

2. Prevalence: In total, 7.8% of patients (17.2% female) with acromegaly and gigantism from the FIPA consortium harbor a *GPR101* mutation [25].

3. Clinical features: The majority of X-LAG patients are delivered at term, with normal birth weights and lengths. They show an earlier median age at the beginning of aberrant development compared to other children who have *AIP* and *GPR101*-negative gigantism (1–1.9 years vs. 16 years) and an increased acceleration in height. The median age at diagnosis is 3.4–4.4 years, with a median height between +3.9 and +5.4 standard deviations. The condition takes an average of 2.6 years to manifest before a diagnosis is made. Based on the data provided by Trivellin et al., Iacovazzo et al., and Beckers et al., the mean age at diagnosis is 4.7 years. There is a female predominance. X-LAG is more commonly linked to hyperprolactinemia than *AIP*-positive gigantism, the levels of prolactin (PRL) being higher in the case of X-LAG. There are no notable differences in the levels of IGF-1 between the two causes of gigantism [24,25,26].

4. Histological findings: The tumors are usually macroadenomas (75%) and exhibit a sinusoidal and lobular architecture, with suprasellar extension, and sometimes extension into the cavernous sinus. The median maximum tumor diameter is 16–18 mm. Of the X-LAG cases, 25% have diffuse gland enlargement indicative of pituitary hyperplasia, either associated or not associated with an adenoma. These are both sparsely and densely granulated adenomas, frequently intermixed with lactotroph cells. Usually, there are two distinct populations of cells, GH- and PRL-positive, and no co-localization of the two hormones. Additionally, follicle-like structures and calcifications are frequently observed. The expression of somatostatin receptors is variable, but generally, SSR2 is constantly present and SSR5 is variably present. In some cases, SSR3 (somatostatin receptor type 3) is present [24,25,26].

5. Genetics: X-LAG, recently recognized as the first endocrine ‘TADopathy’, is caused by constitutive or sporadic mosaic duplications on chromosome Xq26.3. These duplications disrupt the normal chromatin structure within a topologically associating domain (TAD) that typically surrounds the orphan G-protein-coupled receptor GPR101 and protects it from nearby regulatory elements. This disruption leads to the formation of a novel TAD, where ectopic enhancers drive GPR101 overexpression, ultimately causing gigantism [101,102].

X-LAG is transmitted in an X-linked manner. At first, patients with this syndrome were identified as having a duplication on the chromosome Xq26.3, that contained four genes: *ARHGEF6*, *CD40LG*, *GPR101*, and *RBMX*. It was later determined that only the *GPR101* gene would be pathogenic [24,25]. The *GPR101* gene encodes a 508-amino-acid-long protein: probable G-protein-coupled receptor 101 [10]. This protein is highly expressed in the hypothalamus (the arcuate nucleus), the nucleus accumbens, and the pituitary gland (both in the fetal period and in adolescents) [103,104]. GPR101 constitutionally activates the cAMP pathway by stimulating cells to produce the second cAMP in the absence of ligands and activates other G-protein subunits (Gq/11 and G12/13), which can drive GH secretion [104,105]. GNRH (Gonadotropin-Releasing Hormone) neurons are localized in the arcuate nucleus, where a high expression of GPR101 has been observed, which leads to the hypothesis that GPR101 could be involved in the regulation of the GNRH-GH axis [103].

To date, approximately 40 people have been confirmed clinically and genetically to have X-LAG [24,25,26,101,106,107,108,109,110,111,112]. Only seven familial cases have been reported; these were grouped in three unrelated kindreds, and the probands were all male, with an inherited *GPR101* duplication from affected mothers [24,26,108,113].

In cases of a single occurrence in a family, if the proband is female, then the *GPR101* duplication is usually germline, compared to a male proband, where the mutation is usually somatic and with variable levels of mosaicism. In family cases of X-LAG, the *GPR101* duplication is probably germline [27].

Because it is assumed that all females with X-LAG have de novo duplications, the risk for their parents and siblings is low (possible germline mosaicism in parents), but the risk in an offspring is 50%. In cases with a male proband, there are two possibilities. If the proband is a simplex case, then the mother most likely is not affected, but if there is another sibling involved, then the mother is probably heterozygous for the *GPR101* duplication. Only the daughters of an affected male can inherit the duplication, but there have been no such cases so far [26,27].

6. Personalized treatment: None of the patients were able to attain hormonal control with medical therapy alone [24,25,102]. In the latest X-LAG case described by Caruso et al., they noticed that the child, who was only 3 to 4 years old, did not respond well to adult-appropriate doses of first-generation somatostatin analogs during the pre-operative phase. On the other hand, prolactin quickly returned to normal when low dosages of cabergoline were used [102]. In many situations, either drastic or repeated surgery interventions, or the use in conjunction of radiation or Pegvisomant, were required. Despite the presence of SSR2, somatostatin analogs, either alone or combined with dopamine agonists, show poor results. Pegvisomant is more effective in normalizing IGF-1 levels and diminishing the growth rate. Permanent hypopituitarism is observed at a high rate, likely attributed to the necessity of multiple interventions [24,25].

7. Benefits of genetic testing: Microarray-based comparative genomic hybridization can be used to screen for *GPR101* duplications, and, if this does not prove adequate, copy number variation can be examined using droplet digital PCR to find minor duplications and low-level mosaicism [25,107,109]. The penetrance of 100% highlights the importance of genetic testing and the possible application of preimplantation genetic testing in family planning for those who are at risk of inheriting or transmitting the mutation [114].

#### 2.1.3. Unknown Mutations

In approximately 90% of FIPA families, there is no identified germinal mutation. Since pituitary adenomas have a 1 in 1000 prevalence in the general population, it is plausible that the aggregation in certain families is merely coincidental [114].

When contrasting *AIP*-positive and *AIP*-negative FIPA families, the count of affected individuals is notably reduced in *AIP*-negative lineages, indicating lower penetrance [19]; therefore, if *AIP* mutations have been excluded, then no additional genetic or clinical evaluations are advised for the family [114]. Although more evidence is required, several genes (such as *CDH23*—cadherin-related 23 and *IGSF1*—immunoglobulin superfamily member 1) have been proposed as members of the FIPAs and possible generators of GH-secreting PitNETs [28,29]. Also, germline mutations causing loss of function in the peptidylglycine alpha-amidating monooxygenase (*PAM*) gene have been reported in several isolated cases of PitNETs and in one family with pituitary gigantism and need further investigation [30].

### 2.2. Sporadic Acromegaly

#### 2.2.1. AIP

1. General information: *AIP* mutations can be found in individuals negative for family history of pituitary neuroendocrine tumors, who therefore cannot be included in FIPAs. These cases may arise due to spontaneous mutations occurring de novo (which is very rare) [14], inherited mutations with incomplete penetrance (probably the most prevalent mechanism), or mosaicism [114]. They can also appear to be sporadic because of the lack of screening of the relatives. Cazabat et al. demonstrated in their study of *AIP*-positive pituitary adenomas that none of the screened cases exhibited de novo mutations. In each instance, one of the parents was identified as an asymptomatic carrier. The screening of the parents included hormonal evaluation or MRI (magnetic resonance imaging), revealing no evidence of pituitary adenoma in any of them [31].

2. Prevalence: The prevalence is approximately 3.4–5% of all the *AIP*-positive cases [14,19,31,32].

3. Personalized treatment: Sporadic *AIP* mutations are responsible for 8% of the cases resistant to somatostatin analogs [33].

4. Clinical features: Patients with acromegaly and *AIP* mutations are significantly younger than those without *AIP* mutations, with a mean age at diagnosis of 23.5 ± 4.2 years. There is a male prevalence, and the majority of the cases are macroadenomas. Cazabat et al. showed in their study that 83% of the AIP-positive patients had gigantism, indicating that the condition started early in life. On the other hand, gigantism was only seen in 9% of the *AIP*-negative patients [31].

5. Genetics: Detailed information about the *AIP* gene was presented before in the chapter dedicated to FIPA.

6. As for histological findings, we did not find data in the literature for sporadic *AIP* in particular, because they are characterized together with the FIPA *AIP* mutations previously discussed in this article [19,20].

7. Benefits of genetic testing: As *AIP* mutations are known to be responsible for FIPAs, and the exact penetrance of each mutation is not defined, there is a certain need to screen relatives [17,100].

#### 2.2.2. GNAS Complex Locus (GNAS)

1. General information: *GNAS* (previously named guanine nucleotide-binding protein, alpha-stimulating activity polypeptide 1 [22]) is responsible for encoding the stimulatory G protein subunit alpha isoform (Gαi). The proposed mechanism behind tumorigenesis involves the dysregulation of the cAMP PKA signaling pathway, ultimately resulting in hormonal hypersecretion and the disruption of the cell cycle [37].

2. Prevalence: Roughly 30–40% of GH-secreting pituitary adenomas possess a somatic *GNAS* mutation. In fact, the most frequent somatic mutation associated with pituitary adenomas is a gain of function mutation of *GNAS* [34,35,36,37]. There are cohorts that show an even higher prevalence of almost 55% [115,116].

3. Clinical features: There is a distinct GNAS-related phenotype, characterized by older patients with notably smaller, less invasive tumors and a slower growth rate. Some report the mean age at diagnosis as 42.0 ± 1.6 years; others report the median as 41 years (ranging from 34 to 48). Even though the level of IGF-1 is higher at diagnosis, an immediate postoperative decrease in nadir GH levels has been reported [36,38,39].

4. Histological findings: The tumors are usually densely granulated [40,41]. Regarding the proliferation rate, individuals in the mutant group generally exhibit lower Ki-67 indexes, with only 27.0% having a Ki-67 index ≥3%, compared to 47.8% in the *GNAS*-negative group [36]. Higher levels of D(2) dopamine receptors (DRD2s) are expressed by those with *GNAS* mutations [42].

5. Genetics: *GNAS* mutations produced early in the embryonic development stage are the cause of MAS. If the same mutations occur postnatally, then they are responsible for sporadic disorders, such as sporadic acromegaly [43]. Detailed information about the *GNAS* gene and its pathogenic variants is presented in the chapter dedicated to MAS.

6. Personalized treatment: There is a more favorable response to pharmacological (first-generation somatostatin analogs) and surgical interventions when compared to the *GNAS*-negative acromegalic patients [39,40]. The discovery that *GNAS* copy number gain can significantly increase tumor cell proliferation suggests that inhibitor therapy targeting CDK6 (cyclin-dependent kinase 6) might prove effective for PIT1 (pituitary-specific positive transcription factor 1) lineage patients with *GNAS* copy number amplification [117].

7. Benefits of genetic testing: Identifying *GNAS* mutations is crucial, as they are linked to improved responses to surgery and to first-generation SAs. This knowledge enables a more accurate prognosis, potentially allowing for longer intervals between appointments and easing the patient’s life. Moreover, distinguishing between aggressive and milder forms of acromegaly is not only beneficial for the patient’s physical wellbeing but also aids physicians in developing appropriate treatment plans. Higher levels of DRD2 may predict a better response to dopamine agonists [42].

#### 2.2.3. Higher Gastric Inhibitory Polypeptide Receptor (GIPR) Expression

1. General information: There are acromegalic patients in whom glucose develops a paradoxical response of GH (increase). Regarding the definition of a paradoxical GH response following an oral glucose tolerance test (OGTT), there is no agreement, but it may be appropriate to define it as a 20% increase occurring within the first 90 min [118].

2. Prevalence: Overall, 24–27.4% of patients exhibit a paradoxical increase in GH levels following an OGTT [44,45].

3. Clinical features: Some researchers have suggested that acromegaly patients exhibiting a paradoxical GH response pattern appear to have higher concentrations of IGF-1 and tend to have smaller adenomas compared to acromegaly patients without paradoxical GH response [46], but others do not sustain these data, affirming that gender distribution and basal tumor diameter were similar in both groups [119]. The mean age at diagnosis in this group of patients is 44.08 ± 12.37 years, similar to the one without paradoxical GH response (40.97 ± 11.01) [119].

4. Histological findings: Ectopic expression of the GIPR was found in these patients’ somatotropinomas [44,45,120]. There is a predisposition to densely granulated adenomas [46].

5. Genetics: It is now clear that there are no *KDM1A* (lysine demethylase 1A) germline mutations in acromegalic patients, like in gastric inhibitory polypeptide (GIP, alternatively named glucose-dependent insulinotropic polypeptide [10])-dependent primary bilateral macronodular adrenal hyperplasia. Instead, there is reportedly a recurrent loss of chromosome 1p—so-called *KDM1A* haploinsufficiency [44,45]. KDM1A’s interaction with DNA methyltransferases potentially serves as a crucial link between histone modification and DNA methylation, playing a significant role in GH-producing pituitary adenomas [121]. The ectopic expression of GIPR likely results from diminished transcriptional activation and is probably influenced by microamplifications of the *GIPR* gene and abnormalities in DNA methylation. The adenomas that show DNA hypermethylation do not express *GNAS* mutations [120].

In neuroendocrine tumors, GIPR expression may be correlated with an increased proliferative rate and even with metastases development. However, recent studies suggest that the ectopic expression of GIPR might not be the primary cause of tumor development, but, rather, a subsequent occurrence triggered by epigenetic factors [122].

6. Personalized treatment: The response to SAs is more favorable compared to acromegaly without paradoxical increase after OGTT (with the higher rate of remission in response to medical therapy after surgery of 83% vs. 55%), but the risk of developing diabetes after Pasireotide treatment is higher. Additionally, overall treatment response rates are greater (89% vs. 71%) [118,119]. Moreover, the persistent paradoxical GH response following surgery might serve as a biological indicator of residual disease postoperatively. Introducing targeted therapy to antagonize the GIPR on GIPR-expressing somatotroph adenomas could represent a novel treatment approach for acromegaly patients with a paradoxical GH response pattern to OGTT [46].

7. Benefits of genetic testing: The paradoxical GH response to OGTT indicates a lower probability of disease recurrence after initial surgery. As a result, for patients with acromegaly, the pattern of GH responsiveness to OGTT is helpful in predicting long-term results. Also, in the postoperative phase, the persistent paradoxical GH response could make it challenging to differentiate between a genuinely high GH level requiring further intervention (such as medication, a second surgery, or radiotherapy) and a GH level that appears elevated solely due to the glucose load, as it seems to appear in some people without acromegaly. Knowing the higher GIPR expression in such situations could help guide management [46,118,123].

## 3. Syndromic Acromegaly

**Figure 2:**
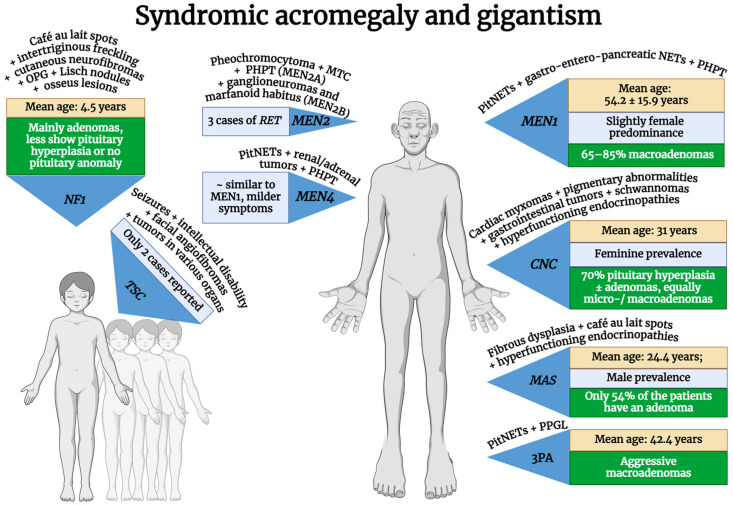
Syndromic acromegaly and gigantism. Abbreviations: OPG—optic pathway glioma; NF1—neurofibromatosis type 1; TSC—tuberous sclerosis complex; MEN2—multiple endocrine neoplasia type 2; *RET*—ret proto-oncogene mutation; MTC—medullary thyroid carcinoma; PHPT—primary hyperparathyroidism; MEN4—multiple endocrine neoplasia type 4; PitNETs—pituitary neuroendocrine tumors; MEN1—multiple endocrine neoplasia type 1; NETs—neuroendocrine tumors; CNC—Carney complex; MAS—McCune–Albright syndrome; 3PA—3P association; PPGL—pheochromocytoma/paraganglioma. The mean age refers to the average age at which acromegaly/gigantism occurs. The sex prevalence and pituitary anomalies refer to PitNETs. Created with BioRender.com (accessed on 14 August 2024).

Syndromes associated with acromegaly include multiple endocrine neoplasia type 1 (MEN1) and type 4 (MEN4), Carney complex type 1 (CNC), McCune–Albright syndrome (MAS), the 3P association (3Pa), and neurofibromatosis type 1 (NF1). In this chapter, we will also discuss the association of acromegaly with less common syndromes, such as tuberous sclerosis complex (TSC) and multiple endocrine neoplasia type 2 (MEN2). A brief overview of the syndromes involving acromegaly is provided in Figure 2.

### 3.1. Multiple Endocrine Neoplasia Type 1 (MEN1)

1. General information: Multiple Endocrine Neoplasia type 1 is a rare autosomal dominant inherited disease that affects the parathyroid, the pituitary, and the neuroendocrine tissue of the gastro-entero-pancreatic system. In 90% of cases, the menin 1 (*MEN1*) mutation is inherited from an affected parent, while the other 10% cases are the result of de novo pathogenic mutations that occur early in embryogenesis [47]. Clinical MEN1 can be identified by having two of these endocrine tumors together or having at least one along with a relative who has already been diagnosed with MEN1 [124]. Penetrance varies with age, beginning at approximately 5 years old and gradually increasing to >94% by the age of 50 years and 100% of all cases by 60 years. The degree of involvement varies depending on the organ: parathyroid involvement is the most prevalent, almost ubiquitous, and frequently the earliest [48,49]. PitNETs are the first manifestation in approximately 20% of MEN1 cases, many of which are diagnosed in childhood and adolescence [125].

2. Prevalence: MEN1’s incidence and prevalence in the general population are difficult to estimate, but the estimated prevalence is between 1 and 3 per 100,000 individuals [48]. Among patients with acromegaly, the proportion of clinical MEN1 varies widely, between 2.9% and 18.5%, but Nachtigall, Guarda et al. performed a large study where it was shown to be 6.6% [50]. On the other hand, pituitary involvement affects 30–40% of MEN1 patients, and a significant proportion of all pituitary tumors (5% to 25%) are due to GH-secreting tumors [50,126,127,128]. In addition, 1.2% of patients without clinical signs of familial history and aged under 30 with sporadic cases of acromegaly were found to have a mutation in the *MEN1* gene [128].

3. Clinical features: Although the association between hyperparathyroidism and neuroendocrine tumors (NETs) is most common in MEN1, the clinical presentation of MEN1, which includes acromegaly, is primarily characterized by the co-occurrence of acromegaly and primary hyperparathyroidism. Less commonly observed is the combination of acromegaly and gastrointestinal NETs. Acromegaly in the context of clinical MEN1 is diagnosed at an older age (mean age, 54.2 ± 15.9 years) compared to isolated acromegaly (mean age, 42.6 ± 14.0 years), usually (in 90.9% of the cases) before or at the same time as primary hyperparathyroidism. There is a slight female predominance (59.1%), like in isolated acromegaly. Additionally, there are no significant differences observed in IGF-1 or prolactin levels at diagnosis or tumor size. In terms of cancers, papillary thyroid carcinoma (13.6% total) is the second most common after breast cancer (23.1% of women) [50,51].

4. Histological findings: In MEN1, 66.7% of adenomas are both GH- and prolactin-positive in immunohistochemistry, compared to isolated acromegaly (76.1%). Overall, pituitary tumors are represented by macroadenomas in 65–85% of MEN1 patients, the proportion being higher than that found in sporadic PitNETs. Locally invasive PitNETs occur in approximately 30% of cases [50,52].

5. Genetics: The *MEN1* gene is located on chromosome 11q13.1; it has 10 exons, is considered to be a tumor-suppressor gene, and encodes menin, a 610-amino-acid-long protein. This scaffold protein regulates gene transcription by coordinating chromatin remodeling and also interacts with numerous transcription factors (such as transcription factor JunD, nuclear factor NF-kappa-B, or SMAD family member 3). It also may be involved in normal hematopoiesis and deoxyribonucleic acid (DNA) repair [10,53,54]. *MEN1* mutations are usually germline, but somatic variants are also found [55]. Sometimes, the two types of mutations do not coexist or do not have the potential to be pathogenic or driver mutations [56].

Inheriting a germline *MEN1* mutation predisposes individuals to develop tumors following a somatic mutation. This latter, typically a point mutation, or, more frequently, a deletion, results in loss of heterozygosity (LOH) in the tumor DNA. Therefore, the normal cells will have both wild-type and mutant alleles of the *MEN1* gene, and the tumor cells, which exhibit LOH (90%), will contain the mutant allele only. If LOH is not detected (10%), then the inactivation of the wild-type allele is probably due to a point mutation or small deletion or insertion within the coding region or the splice sites of the *MEN1* gene [126].

A family history that seems negative can only be verified if molecular genetic testing confirms that neither parent carries the pathogenic variant identified in the proband. For the molecular diagnosis of MEN1 syndrome, single-gene testing, which can detect small intragenic deletions/insertions, missense, nonsense, and splice-site variants, is usually performed first. If no mutation is detected, then the next step is gene-targeted deletion/duplication analysis, which extends the detection of exon and whole-gene deletions or duplications. Comprehensive genomic testing can be utilized in certain situations (where the phenotype is indistinguishable from other tumor-predisposing disorders) [47].

Simplex cases are rare (10%), so the parents of an affected individual have 90% risk of harboring the mutation. Siblings also have a 50% risk if the parents are also affected, and it is important to note that there is a significant clinical variability between members of the same family. The offsprings of a proband also have a 50% risk of inheriting the mutation [47].

6. Personalized treatment: Treatment is similar to isolated somatotropinomas, usually starting with surgery. Additionally, 27.3% of MEN1 GH-secreting adenomas need sellar radiation therapy [50,127,129]. The response to drug therapy is poor, but still has a better prognosis compared to AIP-positive adenomas [57]. Since there are cases of extra-pituitary GH production, it is crucial to rule out ectopic acromegaly in cases of MEN1-related acromegaly to avoid performing unnecessary pituitary surgery on a patient [130].

7. Benefits of genetic testing: MEN1 symptoms can start as early as age five; thus, genetic testing needs to be provided as soon as possible. Patients who have been diagnosed with clinical MEN1 and their first-degree relatives should be offered the opportunity to undergo *MEN1* germline mutation testing. Testing for *MEN1* germline mutations might also be advised for people exhibiting atypical MEN1 symptoms, like multiglandular hyperparathyroidism. Further testing, such as *MEN1* locus haplotype analysis or testing for partial or whole-gene deletion, should be taken into consideration if no *MEN1* coding region mutation is discovered. If an *MEN1* germline mutation is discovered, then the patient should be regularly screened for the development of tumors linked to MEN1. Biochemical screening for pituitary tumors may involve a yearly evaluation of plasma prolactin and IGF-I levels and an MRI of the pituitary every 3–5 years. Hypothalamic pituitary testing should be performed in individuals exhibiting aberrant results in order to further describe the type of pituitary lesion and the eventual presence of pituitary insufficiency. The morbidity and mortality in MEN1 should be lowered through the early identification and treatment of malignant neuroendocrine tumors [126].

### 3.2. Multiple Endocrine Neoplasia Type 4 (MEN4)

1. General information: This rare syndrome is caused by germline mutations of the *CDKN1B* (cyclin-dependent kinase inhibitor 1B) tumor-suppressor gene and is characterized by the association of primary hyperparathyroidism and pituitary, renal, and adrenal tumors. Approximately 5–30% of patients with a clinical picture suggestive of MEN1 do not have positive *MEN1* mutations. In this case, MEN4 syndrome should be considered, even though there are many *MEN1* phenocopies and only 1–3.5% of them are due to loss-of-function mutations in the *CDKN1B* gene [3,40,62,63].

2. Prevalence: MEN4 was first described in 2006 and it is very rare, with <100 reported cases since then [131]. Acromegaly can be found in 7–10% MEN4 kindreds [63].

3. Clinical features: MEN4 overlaps MEN1’s clinical image, but it is characterized by milder symptoms and an older age at onset [63]. Acromegaly in the context of MEN4 is not well defined. Singeisen et al. performed a systematic review of the literature from 2006 to August 2022, describing 65 cases of MEN4, only 4 of which included GH-secreting PitNETs. The age at diagnosis of acromegaly was 5, 30, or 62 years, while one case did not have available data regarding this age [132].

4. We did not find histological findings specific to MEN4 syndrome.

5. Genetics: MEN4 syndrome is inherited in an autosomal dominant manner, with most individuals having an affected parent, with a probably complete penetrance [64]. The *CDKN1B* gene is located on chromosome 12p13.1 [22]. Sporadic tumors (including breast cancer, prostate cancer, neuroendocrine tumors, and others) occurring as single tumors in the absence of any other findings of MEN4 frequently contain a somatic pathogenic variant in *CDKN1B* that is not present in the germline. In these circumstances, predisposition to these tumors is not heritable. *CDKN1B* mutations responsible for MEN4 are germline mutations [65]; de novo mutations exist, but the frequency is not clearly established [65]. *CDKN1B* is a tumor-suppressor gene, and it encodes the protein with the same name. Cyclin-dependent kinase inhibitor 1B protein has an important role in the regulation of cell cycle progression by blocking the switch between G1 and S phase. The tumorigenesis in MEN4 syndrome is probably due to reduced levels of the protein, secondary to proteasomal degradation [10].

The parents of an individual with a pathogenic variant may be affected and diagnosed with the same mutation, but there is also a possibility of parental germline mosaicism. The offspring of a proband has a 50% chance of inheriting the variant. It is important to know that among the same family, there could be various types of MEN4-related tumors because of the reduced penetrance [65].

Since the clinical differentiation between MEN4 and MEN1 or coincidental tumor co-occurrence is hard, molecular genetic testing is indubitably a necessity. Diagnosis is established in an individual with a germline heterozygous pathogenic variant in the *CDKN1B* gene. Single-gene testing is usually performed, and it confirms the variant in 95% of cases by detecting small intragenic deletions/insertions and missense, nonsense, and splice-site variants [65].

A study by Halperin et al. proposed that individual with pathogenic variants in codons 94–96 have a higher risk for primary hyperparathyroidism and pituitary adenomas compared with other variants [133].

The molecular diagnosis of asymptomatic family members is essential for the prompt initialization of surveillance and treatment, and the early detection of potentially malignancies can reduce the morbidity and mortality rate [65].

6. Personalized treatment: There are no standard protocols for therapeutic management in MEN4 PitNETs, though standard therapy is applied. Usually, the goal is to achieve remission by surgery, but, if not, somatostatin analogs, pegvisomant, and cabergoline could be considered. Also, a potential novel therapy targeting p27 might bring benefits, but more data are needed [63].

7. Benefits of genetic testing: Following the standard guidelines, every patient exhibiting clinical signs of MEN1 syndrome should undergo testing for pathogenic variants of *MEN1* and *RET* (ret proto-oncogene). In patients who lack an *MEN1* pathogenic variant but have clinical symptoms similar to MEN1 syndrome, germline mutations in *CDKN1B* should be examined by next-generation sequencing (NGS). Given that MEN disorders are inherited in an autosomal dominant manner, with almost-complete penetrance, genetic testing must be made available to all first-degree relatives of MEN4 patients. More research is required in order to determinate the exact tumor risk associated with MEN4 and to develop a screening protocol, though finding a *CDKN1B* pathogenic mutation should lead to periodical clinical, biochemical, and radiological screening similar to MEN1 patients until new recommendations arise [63].

### 3.3. Carney Complex (CNC)

1. General information: Carney complex is a rare autosomal dominant disease with almost complete penetrance. It exhibits a great range of clinical characteristics affecting the neurological, gastrointestinal, endocrine, dermatological, and cardiovascular systems. CNC is frequently linked to mutations in genes involved in the cAMP-PKA signaling pathway, such as protein kinase cAMP-dependent type I regulatory subunit alpha (*PRKAR1A*) and phosphodiesterase 11A (*PDE11A*) [66,67].

2. Prevalence: The prevalence of the syndrome is estimated at 1 in 200,000 individuals [67]. 10% of the CNC patients developacromegaly [68,69]. This proportion could be even higher, as a recent large study (140 subjects) found that 35.7% of the patients with CNC had GH excess, and 20% had symptomatic acromegaly [134].

3. Clinical features: The characteristic features of the syndrome are cardiac myxomas, mucocutaneous pigmentary abnormalities, gastrointestinal stromal tumors, schwannomas, and endocrine disturbances, usually associated with overactivity (primary pigmented nodular adrenocortical disease, GH-secreting PitNETs, testicular tumors, ovarian cysts/tumors, or thyroid nodules/cancer) [67,70]. In an extensive analysis of the literature, Cuny et al. identified 57 cases of CNC-related acromegaly, with a feminine prevalence (33 women vs. 24 men), with a median age at diagnosis of 28.8 years, which is younger compared to sporadic acromegaly [69]. Tatsi et al. found an even lower median age at diagnosis of 25.3 years [134]. The mean age at diagnosis for *PRKAR1A* patients is 31 years, ranging from 16 to 55 [77]. Acromegaly was the first manifestation of CNC in 4 of the 57 cases. Unsuppressed GH at OGTT was almost constant, but IGF-1 was found to be normal in some cases. If elevated, the mean value was 2.1 ± 1.2 the upper limit of normal [69].

4. Histological findings: Up to 70% of CNC patients develop pituitary hyperplasia alone or before the appearance of PitNETs, which can be responsible for subtle GH axis anomalies, such as high baseline GH or IGF-1, or non-suppressible GH following an OGTT. The proportion of macroadenomas to microadenomas is almost equal, discrepant from other causes of acromegaly, where most of the PitNETs are greater than 10 mm. There is a comparable proportion between GH/PRL mixed adenomas and pure GH-secreting adenomas [69].

5. Genetics: *PRKAR1A* is a tumor-suppressor gene and encodes cAMP-dependent protein kinase type I-alpha regulatory subunit, involved in a variety of cellular functions. This protein controls the majority of c-AMP signaling pathways and regulates the catalytic subunit of protein kinase A; therefore, its loss of function leads to increased endocrine hormone signaling and to the development of tumors [10,54,71]. The *PDE11A* gene encodes a 933-amino-acid-long protein: dual 3′,5′-cyclic-AMP and -GMP (cyclic guanosine monophosphate) phosphodiesterase 11A. The isoform 1 of this protein is present in the pituitary gland, and it is involved in signal transduction by regulating the intracellular concentration of the cyclic nucleotides cAMP and cGMP [10].

CNC pathogenic variants are inherited in an autosomal dominant manner in 70% of cases, while de novo mutations are responsible for the other 30% of cases. Molecular diagnosis is established in individuals with suggestive clinical features by finding a heterozygous germline pathogenic variant in *PRKAR1A* [72]. This is the most common genetic anomaly, being responsible for 99.3% of CNC patients with GH excess. A higher risk of developing acromegaly was observed in patients carrying the hotspot variant c.491_492delTG or a variant that led to no expression of the affected allele [134].

By the age of 50, individuals with a *PRKAR1A* pathogenic variant typically exhibit a penetrance rate exceeding 95% for CNC. Incomplete penetrance of CNC (mild symptoms) has been observed in association with only two *PRKAR1A* pathogenic variants: the splice-site variant c.709-7_709-2delTTTTTA and the initiation-alternating substitution c.1A>G (p.Met1Val) [135]. Exon variants in *PRKAR1A* were found to be linked more often with acromegaly, lentigines, primary pigmented nodular adrenocortical disease, and cardiac myxomas compared to intron variants, while milder phenotypes tend to be more commonly associated with splice-site variants [72].

It is possible for spontaneous abortions to appear in pregnancies with a *PRKAR1A* inactivating variant, and fertility difficulties can appear in males with CNC, but in the rest of cases, the risk of inheriting the mutation in offspring in 50% [72].

Besides *PRKAR1A* and *PDE11A*, other genes are also involved in CNC appearance, like *PRKACA* and *PRKACB*. There is also evidence of one case of acromegaly in a 19-year-old female with CNC caused by a gain-of-function (triplication) mutation in *PRKACB* [136]. 

6. Personalized treatment: In 86% of cases, surgery was performed. While some patients were treated with bromocriptine alone or in conjunction with surgery, one patient had radiation therapy. Furthermore, 2 out of 50 of the patients who had treatment with somatostatin analogs alone or in conjunction with surgery were considered resistant to treatment [69].

7. Benefits of genetic testing: Genetic diagnosis is performed by finding a *PRKAR1A* or *PDE11A* mutation by Sanger sequencing or NGS. Screening family members for possible mutations and clinical manifestations of Carney complex is crucial, even when they do not exhibit symptoms. This is because CNC follows an autosomal dominant inheritance pattern [67].

### 3.4. McCune–Albright Syndrome (MAS)

1. General information: MAS is characterized by the association of fibrous dysplasia, café-au-lait spots, and hyperfunctioning endocrinopathies (Cushing’s syndrome, hyperthyroidism, precocious puberty, and acromegaly) [137].

2. Prevalence: The prevalence of MAS is estimated between 1/100,000 and 1/1,000,000 [138]. Approximately 20–30% of MAS patients have acromegaly [74,137].

3. Clinical features: There is a male prevalence (58–76.9%) [74,137]. At the time of acromegaly diagnosis, the mean age is 24.4 years (ranging from 3 to 64). Accelerated growth indicates somatotroph axis hyperfunction in 85% of pediatric cases, but acromegaly is diagnosed in adulthood in 64% of patients. Almost invariably, acromegaly is linked to skull base fibrous dysplasia. Adult gigantism, defined as a final height greater than 2 m, occurs in 7% of instances. Despite precocious puberty occurring in most MAS patients, the final height is usually normal in adults with MAS and acromegaly, probably due to the association of GH/IGF-1 excess with elevated sex hormones [137]. Of the patients, 32–43% show visual disturbances, usually due to the narrowing of the optic canal rather than due to the compression of the optic chiasm [74,137]. Acute hydrocephalus due to craniofacial fibrous dysplasia in association with a pituitary adenoma is cited as a complication of untreated MAS [139].

4. Histological findings: Only 54% of the patients have an adenoma visible on a computerized tomography scan/MRI, with the others’ GH excess being probably due to pituitary hyperplasia [137]. There is a discrepancy regarding the size of the adenomas, with some reporting 72% being macroadenomas [137] and others reporting 58.3% being microadenomas [140]. In total, 81% of the patients are associated with prolactin secretion [137].

5. Genetics: The *GNAS* gene is located on chromosome 20q13.32, and it consists of 22 exons. It has a complex imprinted expression influenced by parental origin [141]. This locus encodes three different proteins, each of them having a different origin: the guanine nucleotide-binding protein G(s) subunit alpha isoform XLαs (paternally derived), the guanine nucleotide-binding protein G(s) subunit alpha isoform short (biallelically derived), and the neuroendocrine secretory protein 55 (NESP55) (maternally derived) [10,142]. The maternal allele is always responsible for the development of pituitary tumors, as it is the only one expressed in the pituitary gland, thyroid, and ovaries [21,93]. The α subunit of the stimulatory G protein (Gsα) plays an important role in numerous hormonal and signal transduction pathways. After its activation, Gsα also activates adenylyl cyclase, which leads to an increased production of cAMP and cell proliferation in responsive tissues, such as the pituitary gland [75,103,143].

In McCune–Albright syndrome, mutations in the *GNAS* gene can occur in any period of embryonic development and can affect all the germ layers (the endoderm, ectoderm, and mesoderm). This particularity leads to mosaic distribution in mutant cells in different organs and to a variable extension of disease involvement [21,144]. MAS is not inherited. So far, only activating *GNAS* somatic pathogenic variants (occurring in embryogenesis) have been discovered [75].

The most common pathogenic variants (95%) are c.602G>A, p.Arg201His and c.601C>T, p.Arg201Cys. Another mutation, responsible for 5%, of cases is c.680A>T, p.Gln227Leu [75,143,144].

The molecular genetic testing consists of PCR (polymerase chain reaction) or targeted analysis (by sequencing exon 8 and 9 of the *GNAS* gene). The highest sensitivity for the PCR method is obtained by analyzing the affected tissue. The risk for family members is the same as in the general population [75].

6. Personalized treatment: When performed, pituitary surgery is not sufficient, with only 12–24% achieving remission [74,137]. While most patients’ GH/IGF-1 levels were improved by somatostatin analogs, only 30% obtained full control. In most of these unresponsive cases, Pegvisomant produces normal IGF-1 levels, and the 20% which remain unresponsive can be controlled under Pasireotide [76,137]. Further data on pituitary irradiation are necessary, as it appears ineffective, possibly due to the limited follow-up period [137].

7. Benefits of genetic testing: Genetic confirmation is needed to monitor associated diseases in MAS, given its syndromic nature. Currently, there are no data indicating specific genetic variants that are associated with acromegaly or a more severe phenotype. The early diagnosis of associated features should improve prognosis [75].

### 3.5. 3P Association (3Pa)

1. General information: The 3P association refers to the coexistence of pituitary adenoma and pheochromocytoma/paraganglioma (PPGL) in the same patient [77].

2. Prevalence: To our knowledge, 32 cases of 3Pa have been reported to date. Regarding the ones including GH-secreting PitNETs, these are even more rare. *SDHx* (succinate dehydrogenase complex) mutations account for 1.1% of the sporadic PitNETs (3/263 patients) included in the study by Mougel, Lagarde et al. [77]. *SDHx*-related pituitary adenomas are mostly prolactinomas (61%), with somatotrophinomas and nonfunctioning PitNETs accounting for 16% each [78].

3. Clinical features: In the case of *SDHx*, the tumors often present as aggressive macroadenomas [3,78]. Out of the patients who had been reported to have PitNETs carrying *SDHx/MAX* (MYC-associated factor X) mutations, 26% had isolated pituitary adenomas and 19% had a pituitary adenoma first, developing a PPGL after. In comparison with patients with *AIP*/*MEN1* mutations, the mean age of occurrence of pituitary adenomas is older: 42.4 years, ranging from 16 to 72 years [77].

4. Histological findings: To date, the only histopathological findings that can guide 3Pa syndrome are intracytoplasmic vacuoles [77].

5. Genetics: In 3Pa syndrome, multiple genes are involved: *MAX*, *SDHx* (*SDHA*, *SDHAF2*, *SDHB*, *SDHC*, *SDHD*), and *TMEM127* (transmembrane protein 127). De novo pathogenic variants are very rare, and the syndrome is usually inherited in an autosomal dominant manner. *SDHx* genes are tumor-suppressor genes [79]. Each of the *SDHA*, *SDHB*, *SDHC*, and *SDHD* genes encodes a subunit of an enzyme complex, SDH, which is involved in the mitochondrial electron transport chain through the conversion of succinate to fumarate in the Krebs cycle. The *SDHAF2* gene encodes another protein necessary for the flavination of flavoprotein subunit SDHA and for the stabilization of the SDH complex [10]. Pathogenic mutations in *SDHD* and *SDHAF2* exhibit parent-of-origin effects, predominantly leading to disease when inherited from the father. The risk of inheritance in offspring in this case is 50%. Conversely, inheriting such a variant from the mother typically does not pose a risk of disease, although exceptions can arise. There is limited understanding regarding the involvement of *TMEM127* and *MAX* in 3Pa tumorigenesis. *TMEM127* is involved in mTOR (the mammalian target of rapamycin) pathway regulation, while *MAX* controls the transcription of genes [79].

The most common genetic cause is an inactivating germline mutation in *SDHx*. Another identified cause is mutations in the *MAX* gene, which are also germline [3,78]. In the study by Mougel, Lagarde et al., two pathogenic or likely pathogenic variants of *SDH* mutations were found (two in *SDHA* and one in *SDHC*), but all of them were associated with prolactinomas, and none were associated with GH-secreting PitNETs. An *SDHA* c.512G>A p.(Arg171His) heterozygous missense variant was associated with a somatotropinoma, but it is a variant of uncertain significance [77]. Another missense variant, *SDHA* c.869T>C, p.L290P, was recently reported to be associated with a somatolactotroph adenoma, also a variant of uncertain significance, which is likely pathogenic [28]. A *TMEM127* variant (c245–10C>G) was found in 2020, with uncertain significance [145]. As for *MAX* mutations, there are two families known, one with a truncating germline *MAX* variant (c.22G>T, p.Glu8*) and another with a loss-of-function germline variant (c.200C>A, p.Ala67Asp), with only the last one being associated with acromegaly [146]. Another nonsense mutation in the *MAX* gene (NM_002382) [c.223C>T (p.R75X)] was found in a 38-year-old female associated with pheocromocytoma, prolactinoma, and clinical and biochemical characteristics of acromegaly [147].

There are a few proposed phenotype correlations by genes. *MAX* and *TNEM127* germline pathogenic variants are associated with pheochromocytomas, but the latter one can also be associated with head and neck paragangliomas. *SDHA* and *SDHB* germline pathogenic variants are associated with pheochromocytomas and paragangliomas, but *SDHB* ones have an increased risk of metastatic disease, which lead to a higher morbidity and mortality rate. *SDHAF2, SDHC,* and *SDHD* pathogenic variants are mainly associated with head and neck paragangliomas [79].

Hereditary 3Pa is diagnosed in an individual who has either a personal or family history of paraganglioma or pheochromocytoma, along with the identification of a heterozygous pathogenic variant in one of the genes through molecular genetic testing (usually multigene panel tests) [79].

Additionally, 3Pa phenotype can be part of other syndromes involving the pituitary gland, such as MEN1 and, in extremely rare cases, MEN2 [148]. Six cases of 3PA have been linked to germline defects in *MEN1* (four cases) and the *RET* proto-oncogene (two cases), with one case of acromegaly associated with *MEN1* and another with *RET*. It is to be noted that the patients also presented other characteristic features of MEN1 or MEN2 [149].

6. Personalized treatment: Patients with *MAX* mutations require a multimodal treatment approach, combining surgical intervention, SAs, cabergoline, pegvisomant, and radiotherapy, as a single method alone seems insufficient [3].

7. Benefits of genetic testing: It is advised that patients who have PitNETs with a family history of PPGL and/or intracytoplasmic vacuoles in histology should be tested for *SDHx/MAX* genetic disorders [77]. Additionally, PPGL can sometimes be linked to pituitary adenomas through mutations in specific genes, including those associated with *MEN1*. Given this potential overlap, genetic testing should be considered for all patients or families presenting with both PPGL and pituitary adenomas, to identify possible underlying syndromes [148].

### 3.6. Neurofibromatosis Type 1 (NF1)

1. General information: NF1 is an autosomal dominant multisystemic disorder. Café-au-lait macules, intertriginous freckling, multiple cutaneous neurofibromas or plexiform neurofibroma, optic pathway glioma, Lisch nodules, and distinctive osseus lesions (sphenoid dysplasia, anterolateral bowing of the tibia, or pseudoarthrosis of long bones) are the hallmarks of NF1. Learning disabilities and behavioral issues are also common. A diagnosis of NF1 is made when an individual possesses one distinctive clinical feature together with a heterozygous neurofibromin 1 (*NF1*) pathogenic mutation, or two or more of the characteristic clinical symptoms [80].

2. Prevalence: The global incidence of NF1 is 1 in 2500 to 1 in 3000 individuals [54]. Approximately 10% of patients with NF1 present acromegaly or gigantism, typically accompanied by tumors of the optic chiasm [81].

3. Clinical features: GH excess in NF1 is described mainly in children but can also appear in adolescents and adults [82]. The mean age at diagnosis is 4.5 years [83].

4. Histological findings: The MRI images show variability among patients: most patients present with pituitary adenomas, some show pituitary hyperplasia, and some have no pituitary anomalies at all. While a significant proportion of patients also have optic pathway gliomas (OPGs), this association is not consistently observed [82,83].

5. Genetics: NF1 is inherited in an autosomal dominant manner in 50% of cases, with the other half being due to de novo mutations. The mutation rate for the *NF1* gene is among the highest known [80]. The penetrance is complete after childhood, but there are some cases reported with incomplete penetrance, especially in the first years of life. NF1 shows a wide phenotypic variability, even in the same family, where the severity may differ in the presence of the same *NF1* pathogenic variant [84].

The *NF1* gene is located on chromosome 17q11.2 and encodes neurofibromin protein. *NF1* acts as a tumor-suppressor gene and counts 58 exons, and the protein is 2839 amino acids long [10,85]. Neurofibromin regulates the Ras signal transduction pathway and is involved in cellular proliferation and somatic cell division [54]. GH excess has been observed in individuals with NF1, but the exact mechanism is still unknown. The loss of somatostatinergic inhibition from infiltrating OPG into specific pathways has been suggested to dysregulate GH secretion, but this mechanism does not explain all the cases reported [81,82].

Alterations in cell signaling pathways, such as PI3K/Akt/mTOR and Raf/MEK/ERK, seem to be implicated in the pathogenesis of NF1-related sporadic pituitary adenomas. Increased expression and activity of B-Raf- and Akt have been described in pituitary adenomas [81]. Another proposed hypothesis is that certain hormone-secreting pituitary cells are derived from the neural crest, with the same origin as tissues predisposed to neoplasia in NF1 [82].

Individuals with whole-gene *NF1* deletion express somatic overgrowth and large hands and feet, and the development of multiple neurofibromas or plexiform ones occur earlier than in patients with other pathogenic variants [80].

To help characterize the molecular basis of GH excess in NF1, more research is needed, because mutations specific to other syndromes, such as *GPR101* and *GNAS*, have been sporadically observed alongside NF1 [82,150].

6. Personalized treatment: In NF1 children with OPG, GH excess can be reversed, and short-term SA therapy could be sufficient [86].

7. Benefits of genetic testing: Genetic testing is needed in diagnosing the syndrome when only one clinical characteristic is present. However, there are currently no data available regarding the molecular characteristics of NF1 forms associated with acromegaly [80].

### 3.7. Tuberous Sclerosis Complex (TSC)

1. General information: TSC is a multisystemic genetic disorder characterized by the development of tumors that are mostly benign, but are also malignant in various organs, accompanied by the classic triad of seizures, learning disability, and facial angiofibromas [151,152,153].

2. Prevalence: According to recent studies, the population prevalence is approximately 1 in 20,000, and the incidence ranges between 1:5800 and 1:13,520 live births [151,152]. PitNETs were found in eight patients with clinical TSC, but only two of them were somatotrophinomas. However, not all cases of PitNETs had genetic confirmation, and none of the somatotrophinomas were genetically confirmed [87,88,154].

3. Clinical features: The patient presented by Hoffman et al. was 9 years old at diagnosis [88], while details for the second case are not available [87]. Other cases of TSC reported in the literature are associated with localized gigantism, without PitNETs, but this is thought to be because of the shared neural crest origin [155,156,157,158,159].

4. Histological findings: The patient presented by Hoffman et al. presented a macroadenoma [88]. Both cases were associated with prolactin secretion [87,88].

5. Genetics: TSC appears through loss-of-function mutations in the TSC complex subunit 1 and 2 (*TSC1*, *TSC2*) tumor-suppressor genes. *TSC1* is located on chromosome 9q34.13, while *TSC2* is on chromosome 16p13.3 [22,153]. Pathogenic variants in either *TSC1* or *TSC2* disrupt the TSC protein complex, which is a negative regulator of the mTOR pathway, and cause benign tumors to develop in numerous organs. About one third of TSC patients have an autosomal dominant inheritance pattern. The remaining two thirds of TSC genetic etiology are explained by de novo and/or mosaic pathogenic mutations in *TSC1* and *TSC2*. [151]. Although the disease is thought to exhibit complete penetrance, the expressivity of variants can vary significantly among individuals, even within the same family [89].

6. Personalized treatment: The only data available show that the patient presented by Hoffman et al. underwent surgery and radiation, with favorable outcomes [88].

7. Benefits of genetic testing: Despite the generally accepted benefits and guidelines for genetic testing for TSC in general, there are no recommendations for acromegaly or PitNET screening, as these conditions are extremely uncommon in TSC [89].

### 3.8. Multiple Endocrine Neoplasia Type 2 (MEN2)

1. General information: MEN2 is characterized by primary hyperparathyroidism (PHPT), pheochromocytoma, and medullary thyroid carcinoma (MTC). A further division of MEN2 into MEN2A and MEN2B occurs. MEN2A commonly presents with MTC, pheochromocytoma, and PHPT, while MEN2B normally does not have PHPT, but, instead, it presents with colon, lip, and tongue ganglioneuromas, along with a marfanoid habitus and MTC. Almost all cases of MEN2 are due to *RET* mutations [62,160].

2. Prevalence: Approximately 1 in 35,000 people worldwide have MEN2 [61]. PitNETs in MEN2 are extremely rare, and acromegaly have only been described in two cases [58,60]. Recently, a case of acromegaly with an *RET* mutation was described, occurring in the absence of other phenotypic manifestations typically associated with RET mutations [59].

3. Clinical features: The first case was a woman diagnosed with acromegaly at the age of 44, who also developed hyperparathyroidism [58]. The second case was a male diagnosed with acromegaly at 35 years old and with MTC at 40 years old [60]. The third case was a 48-year-old female who was diagnosed with acromegaly and developed a moderately differentiated invasive breast duct adenocarcinoma after 8 years [59].

4. Histological findings: Both the second and third patients’ tumors tested positive in immunohistochemistry for GH and prolactin [59,60].

5. Genetics: MEN 2 is inherited in an autosomal dominant manner, with 100% penetrance. The ret proto-oncogene (*RET*), a tyrosine kinase receptor located on chromosome 10q11.21, has been associated with several cytogenetic alterations that may impact both its extracellular and intracellular domains. These alterations can affect the RET protein signaling pathway. In MEN2, this tyrosine kinase receptor gains function as a result of germline *RET* mutations [22,61].

Somatic *RET* mutations are more frequently associated with specific types of tumors, such as breast cancer and sporadic medullary thyroid carcinoma, while germline *RET* mutations are typically linked to particular syndromes, such as MEN2 [59].

In the pituitary gland, the *RET* proto-oncogene is commonly expressed. Only somatotroph cells express *RET* and GDNF family receptor alpha 1 (*GFRa1*), its co-receptor. RET protein is involved in the downregulation of GH production, encouraging cell survival, and preserving the normal amount of somatotroph cells, though physiological GH levels. In normal pituitary somatotrophs, the RET/PIT1/p14ARF/p53 pathways control apoptosis, while the RET/GDNF pathway manages survival by limiting the expression of p53 and p14ARF (ARF) and modulating PIT1 levels. In acromegaly, every gene involved in the two RET pathways—particularly glial cell-derived neurotrophic factor (*GDNF*)—is specifically enriched. The ARF mRNA expression measured in the tumors at the moment of surgery serves as a prognosis indicator. ARF cut-off values showed whether a patient was responding well to treatment (levels above the cut-off) or whether they showed resistance to treatment (levels below the cut-off) [59,161].

The first article reported a germline mutation in the *RET* proto-oncogene, specifically in exon 13 at codon 791 (TAT/TTT), found in four family members across three generations. Among them, three were presumably unaffected, while a 70-year-old woman presented with hyperparathyroidism and acromegaly. Thus, the germline mutation in *RET* was identified in family members ranging in age from 5 to 70 years, with the woman being the only affected individual [58].

The second article reported only one family member of the index case being affected: his 69-year-old mother. She had both MTC and bilateral pheochromocytoma. The genetic analysis indicated Cys 634 Phe (C634F;TGC-TTC) mutation in both cases, but the index case underwent an extra *MEN1* genetic screening, which revealed the absence of *MEN1* mutations [60].

In the third case, the c.2410G>A (rs79658334), p.Val804Met heterozygous variant in the *RET* gene was identified. This pathogenic variant is reported to be associated with MEN-2A, MEN-2B, familial medullary thyroid carcinoma, pheochromocytoma, hereditary cancer-predisposing syndrome, renal hypodysplasia or aplasia, congenital central hypoventilation syndrome, and Hirschsprung disease. Screening for these syndromes ruled out additional phenotypic features. Family genetic analysis revealed that both the patient’s father and daughter carried the same mutation, but without clinical manifestations [59].

6. Personalized treatment: In the first case, the woman underwent two surgery interventions and was uncontrolled under Octreotide [58]. The second patient was completely cured with transsphenoidal adenomectomy [60], while the third patient had an uncomplete surgery and became biochemically controlled after five years of treatment with Pasireotide in association with Pegvisomant and Cabergoline [59]. One possible treatment for resistant acromegaly (in general) is Sorafenib, an RET inhibitor, which acts by blocking GDNF/AKT serine/threonine kinase 1 survival action through protein kinase AMP-activated catalytic subunit alpha 2 and prevents cell death without changing the RET apoptotic pathway [22,161].

7. Benefits of genetic testing: Although genetic testing for MEN2 is widely recognized for its benefits, there are no specific recommendations for screening for acromegaly or PitNETs, as these conditions are extremely rare in MEN2. Typically, a mutation identified through *RET* proto-oncogene genetic testing classifies an index patient as high-risk, moderate-risk, or low-risk. This classification not only influences the expressivity and penetrance of the disease but also guides the timing of cancer screening and preventive surgery, thus eliminating the need to test for multiple possible mutations in family members [61].

## 4. Conclusions

In conclusion, this review highlights the key role of genetic research in revealing the molecular mechanisms underlying acromegaly. Offering insights from both endocrinological and genetic perspectives, it emphasizes the interaction between genetic factors and hormonal dysregulation involved in the disease. While mutations in genes such as *GNAS* and *AIP* have been well established, further exploration of the genetic landscape holds promise for personalized approaches to diagnosis, treatment, and prognosis. With the continuous innovation in advanced genomic technologies, there is great potential to uncover novel genetic factors contributing to acromegaly susceptibility and disease progression. By continuing to explore the genetic background of acromegaly, we hope to improve patient outcomes and advance our understanding of this complex endocrine disorder.

## Figures and Tables

**Table 1:** Genes associated with acromegaly and their particularities.

Type	Disease/Syndrome	Gene(s)	Location	Inheritance	Penetrance	Prevalence of Acromegaly	Clinical Particularities	Histological Particularities	Treatment Particularities	References
**Isolated**	FIPAs	*AIP* (germline)	11q13.2	AD	20–33%	10–15% of FIPA families	Male predominance; age at diagnosis: median 23 years, mean 23.6 ± 11.2 years	Usually macroadenomas, sparsely granulated, low SSR2 expression	Resistance to I gen SAs; need for radiotherapy, repeated surgery, and multimodal therapy more often	[11,14,15,16,17,18,19,20,21,22,23]
		*GPR101* (germline + somatic)	Xq26.3	X-linked	100%, but 72.2% are de novo mutations	7.8% of FIPAs	Female predominance; earlier age at diagnosis (median, 3.4–4.4 years; mean, 4.7 years)	75% macroadenomas; 25% pituitary hyperplasia; generally, SSR2 is present, but SSR5 is variably present	SAs show poor results, Pegvisomant is more effective	[24,25,26,27]
		*CDH23*	10q22.1	?	?	Proposed as FIPA members, possible generators of GH-secreting PitNETs	?	?	?	[22,28,29,30]
		*IGSF1*	Xq26.1							
		*PAM*	5q21.1							
	Sporadic defects in genes involved in other diseases/syndromes	*AIP* (germline)	11q13.2	?	?	3.4–5% of AIP-positive cases	Male predominance; age at diagnosis: mean, 23.5 ± 4.2 years	Usually macroadenomas, sparsely granulated, low SSR2 expression	8% of SA-resistant cases	[14,19,31,32,33]
		*GNAS* (somatic)	20q13.32	?	?	30–40% of GH-secreting PitNETs	Older patients (age at diagnosis: mean, 42.0 ± 1.6 years; median, 41 years), higher IGF-1	Smaller, less invasive tumors; usually densely granulated; higher levels of DRD2	Improved responses to surgery and to I gen SAs	[34,35,36,37,38,39,40,41,42,43]
	Sporadic higher GIPR expression	*GIPR*	19q13.32	?	?	24–27.4% of acromegaly	Paradoxical increase in GH levels following OGTT; higher IGF-1; mean age at diagnosis: 44.08 ± 12.37 years	Smaller densely granulated adenomas; ectopic expression of GIP receptor in somatotropinomas	Good response to SAs	[22,44,45,46]
**Syndromic**	MEN1	*MEN1* (mostly germline, but somatic variants exist too)	11q13.1	AD	100%	2.9–18.5% of acromegaly	Slight female predominance (59.1%); older age (mean 54.2 ± 15.9 years)	65–85% macroadenomas	Poor response to drug therapy but better prognosis compared to AIP-positive adenomas	[10,47,48,49,50,51,52,53,54,55,56,57]
	MEN2	*RET* (germline, but in some cancers can be somatic)	10q11.21	AD	100%	Three cases of *RET* mutations reported	44 (48-year-old female; 35-year-old male)	Prolactin co-secretion in two cases (data available only for two cases)	?	[22,58,59,60,61]
	MEN4	*CDKN1B* (germline)	12p13.1	AD	Most probably 100%	7–10% of MEN4	Similar to MEN1 but milder symptoms and undetermined mean age	?	Standard therapy	[3,22,40,62,63,64,65]
	Carney complex type 1	*PRKAR1A*	17q24.2	70% AD, 30% de novo	>95%	10% of the CNC	Feminine prevalence. Age at diagnosis: median, 25.3–28.8 years; mean, 31 years	70% pituitary hyperplasia ± adenomas; same proportion of micro- and macroadenomas	Probably normal response: a small proportion of subjects are considered resistant to treatment	[10,22,54,66,67,68,69,70,71,72]
		*PDE11A*	2q31.2							
		*PRKACB*	1p31.1							
		*PRKACA*	19p13.12							
		(germline)								
	McCune–Albright syndrome	*GNAS* (somatic–mosaic)	20q13.32	Not inherited	-	20–30% of MAS	Male prevalence: mean age at diagnosis is 24.4 years	Only 54% of the patients have an adenoma	Poor response to surgery alone; 70% are not fully controlled under I gen SAs; Pegvisomant and Pasireotide have good response	[73,74,75,76]
	3P association	*MAX*	14q23.3	AD	?	Extremely rare	Higher age of onset than AIP/MEN1: mean, 42.4 years	Aggressive macroadenomas, intracytoplasmic vacuoles	Patients with MAX mutations require a multimodal treatment approach	[3,10,77,78,79]
		*SDHA*	5p15.33							
		*SDHAF2*	11q12.2							
		*SDHB*	1p36.13							
		*SDHC*	1q23.3							
		*SDHD*	11q23.1							
		*TMEM127*	2q11.2							
		(germline)								
	Neurofibromatosis type 1	*NF1* (germline, but in some cancers can be somatic)	17q11.2	AD	100%	10% of NF1	Mainly seen in children but can also appear in adolescents and adults; mean age at diagnosis: 4.5 years	Most are pituitary adenomas, some are pituitary hyperplasia, and some have no pituitary anomalies at all; a significant proportion of patients also have OPG	In NF1 children with OPG, GH excess can be reversed, and short-term SA therapy could be sufficient	[10,80,81,82,83,84,85,86]
	Tuberous sclerosis complex	*TSC1*	9q34.13	AD, though expressivity is variable	100%	Only two cases reported	Age at diagnosis: 9 years old (available for just one case)	Macroadenoma (available for just one case); Prolactin co-secretion (two cases)	?	[22,87,88,89]
		*TSC2*	16p13.3							
		(germline)								

FIPA—familial isolated pituitary adenoma; AD—autosomal dominant; SSTR—somatostatin receptor; I gen—first-generation; SAs—somatostatin analogs; ?—unknown; OGTT—oral glucose tolerance test; OPG—optic pathway gliomas; CNC—Carney complex; NF1—neurofibromatosis type 1.
